# Peer-to-Peer Health Communication in Older Adults’ Online Communities: Protocol for a Qualitative Netnographic Study and Co-Design Approach

**DOI:** 10.2196/19834

**Published:** 2020-09-14

**Authors:** Michael Thomas Lawless, Mandy Archibald, Maria Alejandra Pinero de Plaza, Phoebe Drioli-Phillips, Alison Kitson

**Affiliations:** 1 Caring Futures Institute College of Nursing and Health Sciences Flinders University Bedford Park Australia; 2 National Health and Medical Research Council Transdisciplinary Centre of Research Excellence in Frailty Research to Achieve Healthy Ageing Adelaide Australia; 3 College of Nursing Helen Glass Centre for Nursing University of Manitoba Winnipeg, MB Canada; 4 School of Psychology University of Adelaide Adelaide Australia

**Keywords:** aged, chronic illness and disease, long-term conditions, self-management, peer support, social media, online community, netnography, co-design, COVID-19

## Abstract

**Background:**

Online communities provide an environment in which people with similar health concerns can interact and access content that can support the self-management of long-term conditions (LTCs). Recently, the importance of online social networks as sources of health information and social support has been brought into focus with the emergence and widespread societal impacts of COVID-19. Although online communities exist for older adults, little is known about the specific health and self-care topics that older people discuss in such environments and how these relate to users’ support needs and outcomes. A better understanding of users’ needs and peer-to-peer communication in these communities is necessary to inform the design of information and communication technology (ICT) interventions that are relevant to older people and their peer supporters.

**Objective:**

This study aims to use a two-phase, web-based ethnographic (netnography) and co-design approach to explore specific health care and self-care topics that older adults discuss in a UK-based online community and how peer supporters respond to these queries with informational and/or social support and engage with stakeholders to define the needs and requirements for new ICT-based interventions capable of reducing social isolation and facilitating LTC self-management support.

**Methods:**

The first phase of the research will involve a qualitative netnographic analysis of posts in discussion forums in a publicly accessible online community. The second phase will involve co-design workshops with health care consumers (ie, older adults and carers) and service providers to determine the needs and requirements for new ICT-based interventions and digital innovations. Constructivist grounded theory will be used in the first phase; in the second phase, the co-design workshops will be audiorecorded and analyzed thematically.

**Results:**

This research project is in progress. Permission was obtained from the website administrator to use materials from the social media forum; data collection for the first phase began in April 2020. The second phase of the study is expected to begin in late 2020. This study is due to be completed by the end of 2021.

**Conclusions:**

This study is the first, to the best of our knowledge, to combine qualitative netnography with an iterative co-design framework to specify the needs and requirements for new ICT-based interventions. The findings from this study will inform the next phase of the multiphase knowledge translation project and will provide insights into the potential of online peer health communities to reduce social isolation and facilitate chronic illness self-management support and self-care.

**International Registered Report Identifier (IRRID):**

PRR1-10.2196/19834

## Introduction

### Background

Social connections are critical to psychological and physical well-being and are an important component of long-term condition (LTC) self-management support [1-5]. However, for many older adults, reduced mobility, declining health, and separation from family members and friends can make it difficult to access their formal and informal care and social support systems [6,7]. Owing to the COVID-19 pandemic, the health risks of prolonged lockdowns (ie, *stay-at-home* or *shelter-in-place* ordinances given by governments or authorities for enforcing social distancing) have come to the fore as older populations are told to self-isolate for self-protection and to mitigate the spread of severe acute respiratory syndrome coronavirus 2 [8]. Among the health risks, social isolation has been identified as a primary public health concern, amplifying the burden of neurocognitive, autoimmune, cardiovascular, and mental health problems, such as anxiety and depression [8,9]. During the current COVID-19 pandemic, social isolation is expected to disproportionately affect older people, particularly those without close friends or family whose main source of social contact is outside the home [8]. In light of this context, information and communication technologies (ICTs) can play a potentially important role in connecting older adults and their carers to health-related content and supportive social networks, irrespective of their geographical location, physical ability, or the accessibility of health care services [10-13].

Although older people can access health information from their primary health care provider, unmet needs are frequently reported in the literature [1,14,15], with many patients seeking supplementary information and support from online sources [16-20]. Online communities are a source of peer-to-peer communication that enables users to access health-related content and to interact with others for information or social support [11,21]. An online community is defined as “a large, collectivity of voluntary members...whose members share a common interest, experience, or conviction and positive regard for other members, and who interact with one another and contribute to the collectivity primarily over the Net” [22]. These services invite users with common interests or experiences to interact with one another and exchange information and support. By participating in online discussion boards or forums, members can access a network of individuals facing similar situations, learn from others’ experiences or coping strategies, and share views on self-care and self-management activities in relation to specific health conditions [23,24]. Most discussion forums in online communities are asynchronous, enabling users to post and respond to messages at any time and have a hierarchical structure, containing several distinct message boards arranged thematically [25]. Each board contains different threads that consist of an initial post through which a member initiates a new discussion by describing an experience, asking a question, or soliciting advice; other members can then contribute by posting replies.

Older people may find that the content generated and shared by members of online communities differs from and is preferable to the health information available on general websites such as WebMD and the websites of government health or nonprofit organizations [23,26,27]. Content generated and shared by members of online communities may be perceived as more credible and relevant to users’ personal experiences or current symptoms, particularly if the web-based content is readily available and the information provided by health care providers and other offline sources is difficult to access or understand [11,21,24,28]. Moreover, members of online communities may find it more acceptable to receive health information and advice from peers having the same diagnosis, symptoms, or health-related decisions [13,29]. The benefits of participating in online communities in terms of supporting health literacy, resilience, empowerment, psychosocial well-being, and LTC self-management have been documented in the research literature [19,23,30-34]. However, to date, relatively little research attention has been given to the specific health care and self-care topics that older adults discuss in online communities and how peer supporters respond to such queries with informational and social support. This limits opportunities to develop novel digital pathways to facilitate social connectedness, self-care, and LTC self-management support amid the social distancing regulations and increased pressure on health care systems arising from the COVID-19 pandemic.

Previous studies of online health communities have focused on psychological, marketing, and health informatics theories to explain key variables in determining patients’ motivations for participating in online communities and how the information exchanged therein is applied in managing their health [21,35-39]. An important finding from this research is that members of online communities reported general benefits of participation, such as better general LTC self-management capabilities, and specific benefits of participation, such as collaborative problem solving, communication skills and strategies, and approaches to managing negative emotions [24]. A meta-synthesis of qualitative research by Allen et al [28] identified 6 main themes in relation to the negotiation of LTC self-management support in online communities. These themes were then synthesized into an argument centered around 4 key mechanisms for online self-management support: (1) collective knowledge and identification through lived experience; (2) support, information, and engagement through readily accessible online relationships; (3) sociability that extends beyond illness; and (4) online disinhibition as a facilitator in the negotiation of self-management support. From a research perspective, the novelty and significance of this type of web-based research lies in the opportunity to understand and embrace an emerging channel through which individuals act collectively, co-creating understandings and providing peer support outside the remit of traditional health care systems [37]. Such research can also offer a rich source of data on patient-reported outcomes and psychosocial needs [15] that can be collected *naturalistically* (eg, without researcher influence) [40].

Netnography is a web-based ethnographic method for studying cyber cultures “sitting within a broader methodological context of online or virtual ethnography” [41] and a useful exploratory tool for understanding consumer perceptions, experiences, learning, and behaviors in online social networks and communities [42-45]. Netnography studies apply naturalistic, multimethod, and multimodal ethnographic approaches to technologically mediated behaviors and interactions in online social networks [42,43]. Studies of online communities are often less obtrusive, less resource intensive, and more flexible than traditional ethnographic approaches and have been combined with methods such as social network analysis, interviews, and surveys [45,46]. Although studies of online communities have provided insights into consumer cultures and behaviors necessary to inform consumer services, research, and practice [46], few studies have used netnography to research online health communities, specifically for older adults [47-49]. This can partially be explained by the initial presentation of netnography as a marketing research technique [42]. As a result, it has been used predominantly in studying web-based brand communities in connection to older people’s leisure and entertainment [50,51].

Within the fields of marketing and consumer behavior, netnography has been used to examine topics such as consumer identity (eg, identity construction), electronic word of mouth (eg, peer influence), consumption experiences (eg, experience creation), co-creation (eg, product development), and various aspects of online community participation (eg, online cultures, consumer learning) [44-46]. Although netnography has numerous promising applications, it has seen limited use in health care research, with earlier studies exploring topics such as eating disorders [52], breastfeeding [53], alcohol-related problems [54], codeine addiction [55], and the relationship between sexuality and well-being in older adulthood [56]. Moreover, although netnography offers several benefits over offline methods, particularly with regard to the ability to collect timely and continuous naturalistic data, the approach is yet to be utilized to explore the needs and concerns that older adults discuss in online communities in relation to LTC self-management and self-care as a starting point for health research co-design.

### Aims

**Figure 1:**
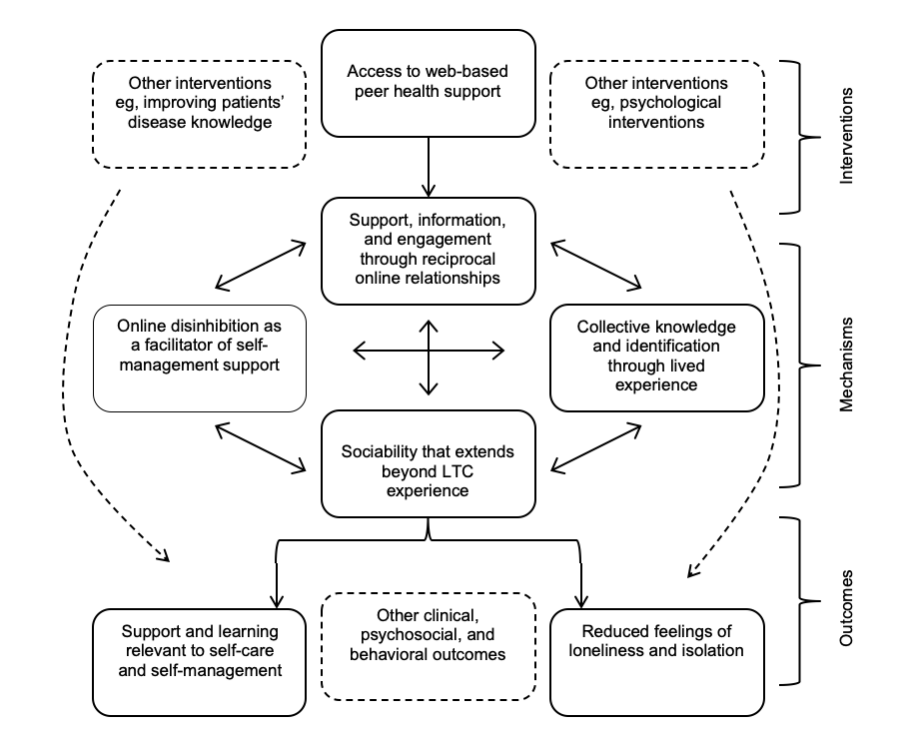
Theoretical model of pathways (logic model). LTC: long-term condition.

This paper describes the protocol for the first 2 phases of a knowledge translation and innovation project called AgeiNg in TechnologIcaLLy mEdiated Spaces (ANTILLES). Broadly, concepts of relational autonomy [57], relationship-centered care [58], apomediation theory (ie, the theory that peer supporters are pivotal in guiding individuals to relevant and credible health information) [59], and a positive psychosocial model of health and well-being in older adulthood [60] underpin this project. To help clarify initial causal assumptions, we depicted possible intervention pathways as a logic model (Figure 1). The model is based on a review by Allen et al [28] on LTC self-management support in online communities, which states that social ties formed in online communities can provide a basis for the performance of relevant self-management work and improve an individual’s experience of living with an LTC.

The specific objectives are as follows:

1. Netnography component: explore the health and self-care topics that older people discuss in relation to LTC self-management and self-care in a publicly accessible online social media site and discussion forum.
2. Co-design component: identify and engage with stakeholders to define the needs and requirements for new ICT-based interventions to reduce social isolation and facilitate LTC self-management support for older adults living independently.

## Methods

### Study Design

We aim to use a two-phase approach, beginning with an in-depth qualitative netnographic study followed by a series of co-design workshops with health care consumers and service providers to synthesize design materials and propose novel ICT interventions.

#### Phase 1: Qualitative Netnographic Study

##### Data Collection

We will identify and analyze posts from online forums on a publicly accessible UK-based entertainment and lifestyle website and social network explicitly targeting older people. To identify the website, we searched Google using the advanced search function for online communities for older adults using combinations of keywords and Boolean operators, including “older*” OR “elder*” OR “senior*” OR “retiree*” AND “discussion*” OR “message board*” OR “forum*” OR “chat room*” AND “health.” Websites were initially identified for inclusion in the study if (1) a stated objective of the website was to provide a platform for peer-to-peer communication among older adults or seniors or retirees about health-related topics, for example, through the hosting of chat rooms, email distribution lists, forums or message boards, and other interactive applications; (2) the website was in English and the materials posted by the users were publicly available and/or the users agreed that their information was nonconfidential and nonproprietary; and (3) the website was established (ie, operating for ≥3 months) and currently active (ie, forums are active and updated daily). The final host website and online community were selected according to several criteria of relevance, representativeness, heterogeneity, substance and critical mass of participants, activity and interactivity, data richness, and experiential features [42].

Textbox 1 provides a summary of the characteristics of the online discussion forum and the website’s terms and conditions. Following earlier netnographic research [52], the name of the website was removed to protect the privacy of forum users. The website was chosen as the forum host as it is a popular website that presents posts and associated conversational threads publicly, thereby minimizing any ethical concerns related to *lurking* on online discussion forums. To locate relevant posts, we will access the online community forum and screen the *general health* discussion board for the 200 most recently active forum threads with posts including keywords generally related to older adults’ self-care and self-management of LTCs in daily life. For the purpose of this study, we draw on the definition by Grady and Gough [61] of *self-management* as “the day-to-day management of chronic conditions by individuals over the course of an illness”; *self-care* is defined as “tasks performed at home by healthy people to prevent illness, rather than merely managing existing illness.” In addition, we will refer to the World Health Organization’s (WHO) International Classification of Functioning, Disability, and Health framework and coding system to assist with the identification of posts related to discrete self-care or self-management tasks and environmental factors [62]. A record will be kept of the full URL for each thread that is downloaded so that it can be located again if it becomes necessary to refer back to the original webpage during data analysis or subsequent revisions before publication [25].

**Description of website and online discussion forum features.**

Forum and website features

- The website hosts online forums on a range of topics, including education, travel, lifestyle (eg, gardening), health, finance, travel, and technology
- The website maintains an active presence on various social media platforms, including Facebook, Twitter, Instagram, and YouTube
- The forum is located within a UK-based entertainment and lifestyle website and social network for adults aged >50 years
- According to the forum’s fact page, at the time of writing the manuscript (April 2020), the forum hosted 2051 topics with at least 22,201 posts
- Forums hosted on the website are publicly accessible for reading and posting via registration
- Forums support asynchronous discussion among users on conversational threads

User features

- Individuals using the discussion forum are known by a username only and the site does not enable users to contact each other privately
- Once users are registered with the site, they are solely responsible for all use and protection of the confidentiality of any user identification and password that they have selected or have been assigned for their access of the site
- User information is defined per the terms and conditions as “any information you provide to us or other users in relation to the service including the forums, blogs, advertising, selling, listing, buying or feedback processes, your postings on the message boards and any other content that you post on the Site”
- Users are solely responsible for their information; the website states that they act as a conduit only for web-based information, comments, advertising, distribution, and publication of users’ information
- Any materials that users upload to the site will be considered nonconfidential and nonproprietary; the website has the right to use, copy, distribute, and disclose to third parties any such material for any purpose

##### Data Analysis

We will use Import.io, a data integration platform that enables automatic extraction of website contents, and download the messages into a Microsoft Excel workbook for storage and management. All textual and graphical materials will then be imported into the qualitative data analysis software package NVivo 12 (QSR International). In line with earlier qualitatively driven netnographic studies [56], we will analyze the data using a constructivist grounded theory approach [63] and elements of situational analysis [64], which extends the grounded theory to include different kinds of *maps* to explore differences and conceptual relationality. The analysis will move through 4 iterative stages: (1) open coding, (2) focused coding, (3) axial coding, and (4) theoretical coding. Extensive memo writing will accompany each step of the analysis. First, preliminary post-by-post and line-by-line open coding by 2 independent analysts across an initial subset of posts will generate a flexible coding framework, which will be iteratively revised as new codes are identified. This phase of the analysis will be concerned with inductively identifying, naming, categorizing, and describing phenomena, concepts, and properties of the data set. Focused coding will then be undertaken to synthesize and filter the preliminary codes or labels down into the most frequent and meaningful initial codes with regard to the research aims and questions. Then, we will conduct axial coding to develop a logical, coherent structure based on the identified codes and the relationships among codes (categories and properties). This process will result in the construction of a working, ordered, and semantic situational map [64]. Finally, we will undertake theoretical coding to refine and name core or superordinate categories, that is, higher-level categories subsuming within them the underlying characteristics of the phenomena of interest and the core concerns of the participants. Throughout this process, we will refer to analytical notes and memos containing reflections on emerging concepts and categories and undertake a constant comparison between focused coding. In our presentation of the results, quotations will be reproduced verbatim, retaining original spelling and grammatical errors, emoticons or emoji, and formatting. We will replace usernames with unique alphanumeric identifiers (eg, the third participant in the second thread of forum 1 will be identified as F1-T2-P03) and apply these to all associated data, including analytic memos, images, and other saved files. NVivo will be used to conduct digital open and focused coding. We will combine this approach with manual methods (eg, note taking, sticky notes, large format display boards) to facilitate a constructivist approach to generate the grounded theory [65]. Mindful of the privacy of forum users, we will not seek respondent validation of the study findings. The anonymous nature of online forum profiles may make it difficult to obtain complete and accurate sociodemographic information about forum users. Although it is possible to derive some personal information (eg, age, gender, marital status) from the posts, this information is not guaranteed to be accurate.

##### Rigor

We will employ an observational approach to netnography, comparable with qualitative studies of naturalistic interactions in online discussion forums [40]. A *passive* researcher role was deemed most appropriate for the exploratory phase of the research because of its emphasis on exploring the aspects of generating an understanding of online cultures, knowledge exchange, peer interaction, and learning to inform knowledge translation and co-design of digital solutions. Nonparticipatory (passive) netnographic approaches have been criticized because of concerns regarding privacy and the lack of opportunity for researchers to conduct their research in ways that directly contribute value to online communities [66,67]. To overcome this limitation, we will record our personal reactions as reflexive field notes while continuing to gain familiarity with the language and practices of the online forum, seeking further stakeholder input in the next phase of the study to clarify understandings or meaning and to contextualize the research findings to the areas of application. Subsequent co-design workshops with stakeholders will provide further context grounded in the diverse experiences and perceptions of current and potential users of online health communities.

We will refer to the Consolidated Criteria for Reporting Qualitative Studies checklist [68] to guide the documentation and reporting of our findings in consideration of credibility, dependability, and transferability. We will demonstrate credibility and dependability by collecting data over a period of 4 to 6 months and by detailing our research processes through an audit trail of methodological and interpretative decisions at each stage of the analysis. We will enhance transferability by describing in detail the original context of the research (ie, documenting characteristics of the website) and providing a detailed account of the processes and nature of the data collected.

#### Phase 2: Co-Design Workshops

Following the netnographic study, we will engage with stakeholders in a series of co-design workshops to (1) discuss the findings of the netnography and deepen our understanding of the perspectives of older people, carers, and service providers toward opportunities and limitations of participating in online social networks for LTC self-management support and (2) define the needs and requirements for new ICT interventions to address social isolation and support LTC self-management. We will use an iterative co-design approach [69,70] employing aspects of experience-based co-design (EBCD) [71] and drawing on techniques applied successfully in previous health technology co-design [72,73]. This approach generally involves stakeholders (eg, staff, patients, and family carers) reflecting on their experiences of using a service or product to collaboratively identify priorities for improvement and to suggest modifications. Co-design workshops are increasingly used in participatory design to help developers and stakeholders share perspectives, and such approaches have been used widely in health service redesign initiatives [69,70]. Co-design principles have been applied in technology-oriented research to ensure that technologies and the services in which they are embedded evolve together, grounded in the needs and experiences of consumers who are engaged in the design process [74]. The ability to incorporate user narratives through stories and the use of specific scenarios can provide a focus for communicating design concepts and how they might be used [75,76].

##### Sample and Procedure

We will conduct 3 co-design workshops with stakeholders, including health and social care providers, carers, and older adults independently living (aged ≥65 years) with lived experience of managing an LTC (assessed using a demographic survey). We anticipate that approximately 8 to 10 people will attend each workshop (number of attendees, N*=*15-30). In addition to these end-user and user community representatives, we identified the following stakeholders for inclusion in our wider design deliberations: (1) project team members, university staff investigators in the research project; (2) external stakeholders, academic clinicians supporting the research; and (3) solution domain experts, independent university staff and technical staff with experience in using or developing ICTs for older adults. Workshop participants will be recruited through the research team’s established network of academic clinicians and a formal process facilitated by a consumer organization working with older South Australians. Participants will be representative of a range of health conditions, sociodemographic characteristics, and experiential, gender, and ethnic diversity. All workshop participants will have some (direct and indirect) experience of using or assisting someone to use ICTs for health-related purposes. We will only include stakeholders who are able to provide informed written consent and communicate sufficiently well in English, as we cannot guarantee the presence of an interpreter during the workshops. The workshops will be facilitated through a South Australian research and product development center co-located with Flinders University that specializes in involving users in co-design and innovation initiatives that respond to global population aging. Remote videoconferencing will be made available in the event of continued COVID-19 disruptions [77].

The aim of the first 2 workshops, conducted with consumers and service providers separately, is to identify goals, define problems, and determine the assumptions to be tested in relation to the following 4 broad questions:

1. What is lacking in the current digital services for older people with LTCs living in the community who experience social isolation?
2. What do we want the solutions to ideally achieve?
3. How can we imagine the solutions failing?
4. Who will be involved in using the solutions?

These questions to be addressed were informed by previous community-based participatory research, including co-design workshops [69]. In the third joint workshop, representatives from both groups will be brought together to discuss solutions and prioritize functions and content. At the beginning of workshops 1 and 2, we will give a short presentation to give background information about the research. We will then screen a brief *trigger film* to each group, derived from our earlier qualitative research [78], to stimulate discussion and support the identification of improvements grounded in authentic consumer experiences. Although conventional EBCD specifies that films can be created with service users and providers at the sites of delivery, our use of the trigger film enables a more efficient process and may also be less threatening to health care providers than appearing to critique existing services [71,79]. Then, drawing on the future workshop approach in participatory design [72], in the initial ideation workshop, participants will discuss existing technologies and services and propose their own imagined solutions, considering features of the existing services that could be adapted, expanded, or repurposed. In the following session, these ideas will be presented in the initial prototypes for critique and collective refinement (Table 1). To help older people engage more authentically and democratically in the co-design process, we will employ visual aids (eg, flow diagrams, card prompts) and various facilitated interactive tasks to help focus attention and facilitate discussion on specific aspects of the design [73,80,81].

**Table 1:** A summary of the co-design workshop procedure and materials.

Characteristics	Workshops 1 and 2	Workshop 3
Sample and procedure	Older adults and carers only participate in initial ideation workshop (workshop 1) to discuss materials and propose solutions Service providers only participate in initial ideation workshop (workshop 2) to discuss materials and propose solutions	Joint workshop bringing representatives from both groups together to discuss synthesized materials and critique prototypes
Materials	*Trigger film* is screened at the beginning of each workshop to stimulate discussion grounded in authentic consumer experiences, followed by persona Persona to be used to provide a shared focus for identifying solutions	Discussion of scenarios derived from synthesis of outputs from workshops 1 and 2 Critique and refinement of specific intervention suggestions
Outcomes	Clarification of goals, problem definition, and assumptions to test Identification of priorities to be discussed in subsequent sessions Generation of scenarios describing prototype interventions	Refinement of initial ideas captured in scenario materials into specific solutions Summary of key issues arising from each workshop in joint display

A secondary aim of this component of the study is to explore whether the design materials presented and developed in the workshops could be utilized to provide a synthesis of key research findings. We will develop a *persona*, intended to be an archetypal narrative description of potential users of a product or service, that will be used to reflect the key characteristics or experiences that need to be taken into consideration when proposing improvements to the current services [82]. To help generate a persona that adequately synthesizes existing research into carers’ and older adults’ perspectives, we draw on the findings from phase 1 of the study and systematic reviews of community-dwelling older adults’ care and support needs [1,14,83]. We will also refer to guidance on developing vignettes or *composite narratives* (ie, an overarching narrative reflective of core aspects of diverse patient experiences) [84] to explore complex public health issues in qualitative research [85]. In line with the prototyping approach used in future workshop methodology, we will then use suggestions raised in the initial workshop to generate *scenarios* [86] describing potential interventions in practice. As scenarios involve action-based narratives, they are more suited than personas for encouraging consideration of the acceptability and feasibility of the proposed solutions and their implementation in the local context [71]. Before workshop 3, the suggestions from workshops 1 and 2 will be synthesized into prototype interventions. By the end of the third workshop, the initial ideas captured in the scenario materials will be refined into specific solutions.

##### Data Analysis

We will obtain consent from all workshop participants to be audiorecorded for research purposes. These recordings will be professionally transcribed and analyzed inductively and thematically using a constant comparative method [87]. Using this approach, transcripts and reflective field notes taken during and immediately after the workshops will be first broken down into concepts and then grouped into categories to summarize key issues arising from each workshop. The authors will review field notes and share their impressions after each workshop so that emerging issues can be discussed and explored in subsequent sessions. Data will then be combined, integrated, and presented visually in a joint display [88] to provide a summary of emerging themes for older adults, carers, and service providers. The results of the netnography will also be presented in an information visualization (eg, an infographic) to help communicate the research findings to diverse audiences.

##### Quality Validation

Owing to the exploratory nature of this research and its focus on emergent co-creation through multistakeholder engagement, we did not prespecify any specific interventions or outcome measures. Although such prespecification is a quality feature in conventional study protocols, it could be considered inappropriate and potentially counterproductive in co-design research [89,90]. This is because, by definition, the specific intervention and its application in the local context are yet to be determined. To ensure credibility and relevance, we plan to codetermine the nature and delivery of the intervention and how its outcomes are measured with stakeholders while concurrently developing research capacity in community partners, establishing program governance, building trust, and working through potential disagreements. In co-design and implementation research, these processes are considered to be mutually reinforcing [89]. Finally, we acknowledge that compromises may have to be made to the proposed methodology to preserve relationships and partnership synergy and to improve impact.

#### Ethical Considerations

Ethical approval was obtained from the Flinders University Social and Behavioral Research Ethics Committee (project number 8559). In phase 1, as the identities of users are unknown (people posting on the website are known only by their username), we were unable to obtain informed consent from individual users to participate in the research. Consistent with contemporary guidelines and ethical codes of conduct for conducting web-based studies [91,92], the first author approached the website via email and obtained permission in October 2019 from the website to use their data, including any text (discussion forum posts and replies) and accompanying visuals for the project period. To enhance transparency, we will advertise and explain the research on the website, Facebook page, and Twitter feed of the website before commencing the study. To further protect the privacy of website users, we will remove terms, images (if attached in forum posts), and phrases that could identify users, their health care providers, and/or carers.

## Results

Data collection for the first phase of the study began in April 2020, approximately 100 days after the WHO was notified of the first cases of COVID-19 in China’s Hubei province [93]. We plan to collect data (ie, forum posts and responses in relevant discussion threads) continuously until October 2020 using observational netnography techniques. The second phase of the study will commence in late 2020 and will be completed by the end of 2021.

## Discussion

Understanding the content and functioning of peer-to-peer interaction in online health communities is critical to the development of ICT-based interventions that are credible, relevant, and meaningful to community-dwelling older people and their supporters. To our knowledge, this study is the first to apply qualitative netnographic and co-design methods to address LTC self-management support for older adults. This study has several outcomes and implications. First, as one of the few studies of older adults’ peer-to-peer health communication in online communities [11,23,48,56], this study will generate practical and potentially transferrable knowledge about the types of health and self-care support that older adults seek and receive from peers on the web and how other users respond to such queries. These insights could be used in future research to inform the design of peer-based web-based interventions and health communication campaigns involving strategic and targeted messaging [94,95].

Second, this study will offer additional learning about how the aspects of EBCD can be integrated with other techniques successfully applied in health technology co-design. One example in this study is the future workshop method, which provides an *early-stage option* [71] for initial idea generation, critique, and refinement, preceding and informing technology coproduction. The use of design materials (ie, persona and scenarios) provides a vehicle for translating research knowledge into reusable and accessible consumer experience resources [84]. Although the selective adoption of multiple co-design approaches and techniques originating from different fields of research can pose a challenge to fidelity, we have attempted to delineate and justify the chosen elements from each. In addition, we acknowledge that the proposed workshops are not a substitute for the need for continued engagement with health care consumers, service providers, and technology industry representatives to fully develop and formally evaluate the intervention.

Third, the study will provide insights into the potential of ICT-based interventions involving open and autonomous online communities (ie, communities created and used mostly by members of the public), which has been identified as “an area in need of and ripe for future study” [15]. For example, social media–based interventions, including forum moderation aimed at stimulating discussion, correcting misinformation as needed, and protecting user safety, might influence the trajectory and quality of web-based peer interaction and enhance users’ self-care confidence and capabilities. Clinical applications could include health professionals’ vetting of and referral to online social media–based peer health groups among their older patients and their carers as a supplementary source of support and/or for patient outreach and extended care [15,23]. Such an approach could extend the benefits of online community participation to the broader population of community-dwelling older adults, while mitigating misgivings about safety and misinformation. This is particularly relevant given the finding that users of online health communities frequently receive support from peers in relation to their unmet needs that they are unable to obtain through their interactions with health care providers and/or their informal carers [15]. Finally, the findings from this study could inform the development of more purposeful design interfaces and educational strategies to address older people’s social isolation and support LTC self-management and self-care in the contexts of limited formal and informal (in-person) care support, during and after the COVID-19 pandemic.

### Conclusions

As countries worldwide grapple with the unparalleled challenges caused by the COVID-19 pandemic, social isolation among community-dwelling older people and the impact of interrupted care and support on LTC management are being recognized as pressing public health concerns [8]. The proposed study addresses this issue by exploring online communities as a medium of peer-to-peer health communication and by engaging with stakeholders to collaboratively develop a novel ICT-based intervention to provide informational and social support networks. The findings from this study will inform the next coproduction and evaluation phase of the ANTILLES project and will have implications for digital health promotion related to social support, LTC self-management and self-care, and peer education and support strategies.

## Abbreviations

ANTILLES: AgiNg in TechnologIcaLLy mEdiated Spaces
EBCD: experience-based co-design
ICT: information and communication technology
LTC: long-term condition
WHO: World Health Organization
